# Unique Biological Activity and Potential Role of Monomeric Laminin-γ2 as a Novel Biomarker for Hepatocellular Carcinoma: A Review

**DOI:** 10.3390/ijms20010226

**Published:** 2019-01-08

**Authors:** Hiroshi Yasuda, Masatoshi Nakagawa, Hirofumi Kiyokawa, Eisaku Yoshida, Toru Yoshimura, Naohiko Koshikawa, Fumio Itoh, Motoharu Seiki

**Affiliations:** 1 Division of Gastroenterology and Hepatology, St. Marianna University School of Medicine, Kawasaki 216-8511, Japan; h2kiyokawa@marianna-u.ac.jp (H.K.); fitoh@marianna-u.ac.jp (F.I.); 2 Division of Cancer Cell Research, Kanagawa Cancer Center Research Institute, Yokohama 241-8515, Japan; Masatoshi.Nakagawa@abbott.com; 3 Diagnostics Division, Abbott Japan Co., Ltd., Chiba 270-2214, Japan; Eisaku.Yoshida@abbott.com (E.Y.); Tohru.Yoshimura@abbott.com (T.Y.); 4 Division of Cancer Cell Research, The Institute of Medical Science, University of Tokyo, Tokyo 108-8639, Japan; mseiki@ims.u-tokyo.ac.jp; 5 Faculty of Medicine, Institute of Medical, Pharmaceutical & Health Science, Kanazawa University, Takara-machi, Kanazawa 920-0934, Japan

**Keywords:** hepatocellular carcinoma, monomeric laminin-γ2, biomarker, α-fetoprotein, prothrombin induced by Vitamin K Absence II, surveillance

## Abstract

Laminin (Ln)-332 consists of α3, β3, and γ2 chains, which mediate epithelial cell adhesion to the basement membrane. Ln-γ2, a component of Ln-332, is frequently expressed as a monomer in the invasion front of several types of malignant tissues without simultaneous expression of Ln-α3 and/or Ln-β3 chains. Moreover, monomeric Ln-γ2 induces tumor cell proliferation and migration in vitro. These unique biological activities indicate that monomeric Ln-γ2 could be a candidate biomarker for early cancer surveillance. However, the present immune method for monomeric Ln-γ2 detection can only predict its expression, since no antibody that specifically reacts with monomeric γ2, but not with heterotrimeric γ2 chain, is commercially available. We have, therefore, developed monoclonal antibodies to specifically detect monomeric Ln-γ2, and devised a highly sensitive method to measure serum monomeric Ln-γ2 levels using a fully automated chemiluminescent immunoassay (CLIA). We evaluated its diagnostic value in sera from patients with several digestive cancers, including hepatocellular carcinoma (HCC), and found serum monomeric Ln-γ2 to be a clinically available biomarker for HCC surveillance. The combination of monomeric Ln-γ2 and prothrombin induced by Vitamin K Absence II (PIVKA-II) may be more sensitive for clinical diagnosis of HCC than any currently used combination.

## 1. Laminin-332

**Figure 1:**
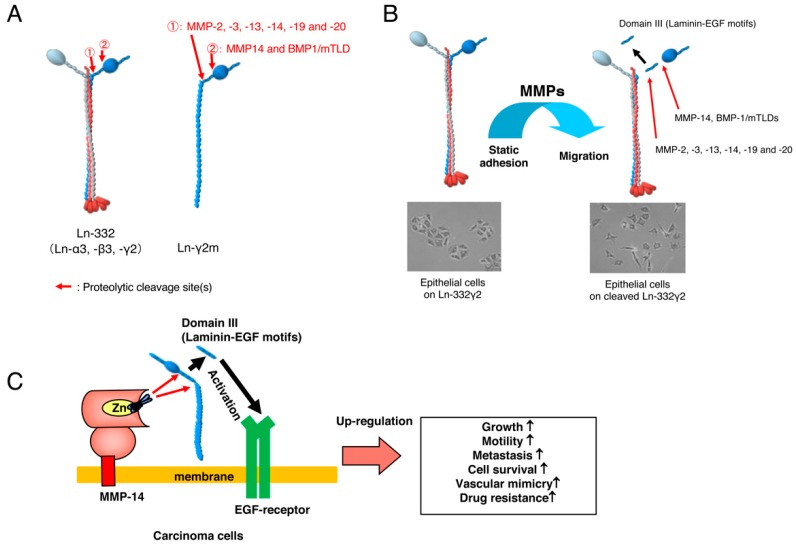
(**A**) The γ2 chain of Ln-332 trimer and monomeric Ln-γ2 chains (Ln-α3 chain: red, Ln-β3 chain: grey, Ln-γ2 chain: blue). Proteolytic processing sites of γ2 chain by MMPs (①) or MMP-14 (②) (**B**) Proteolytic processing of Ln-γ2 chain occurs at cell surface for subsequent regulatory cellular functions (red arrows). Proteolytic processing of γ2 chain converts Ln-332 function from static adhesion to migratory substance. (**C**) Membrane type-1 matrix metalloproteinase (MMP14) cleaves Ln-γ2 chin at both sites and releases the Domain III (laminin-EGF motif) from cancer cells (red arrows), resulting in upregulation of malignant progression of tumor cells through EGFR activation. Figure 1B,C are modified from Koshikawa et al. [8], and Shenk et al. [9].

Extracellular matrix (ECM) proteins in basement membranes (BMs), such as collagen and laminin, play an important role in cell-cell adhesion to maintain epithelial structures in vitro [1]. Among these, Ln-332 (formerly called laminin-5) is a major macromolecule in the epithelial BMs and comprises of three polypeptide chains: Ln-α3, -β3, and -γ2 (Figure 1) [2,3]. In normal epithelium and cancer tissues, heterotrimeric Ln-332 exhibits dual functions of adhesion and migration, and plays important roles in maintaining the static epithelial structure and epithelial cell turnover. These physiopathology functions of Ln-332 are critically regulated by interaction with integrins as a laminin receptor. Integrins α3β1 and α6β4 are a major ligand for Ln-332 and bind the C-terminal domain of Ln-α3 chain, named LG1-3, to promote cellular adhesion and migration [3,4]. Moreover Ln-332 is essential for anchoring epithelia to BMs, and its interaction with integrin α6β4 causes the assembly of hemidesmosomes and immobilization of epithelial cells onto the underlying BMs [5].

So far, it has been reported that many proteases are involved in the processing of each Ln-332 chain [6]. Interestingly, Giannelli et al. reported for the first time that proteolytic processing of the γ2 chain of Ln-332 stimulates mammary epithelial cell motility [7]. Proteolytic cleavage of the γ2 chain of Ln-332 trimer is crucial to convert its static form to a migratory form, since proteolytic Ln-γ2 fragment, Domain III, acts as an EGF-receptor (EGFR) ligand and promotes cell motility [8,9]. Besides integrins, the short arm of Ln-γ2 chain binds a transmembrane heparan sulfate proteoglycan, syndecan-1 and regulates cellular adhesion and migration through Ln-332 by suppressing integrin β4 phosphorylation (Figure 1) [10].

## 2. Monomeric Laminin-γ2

**Figure 2:**
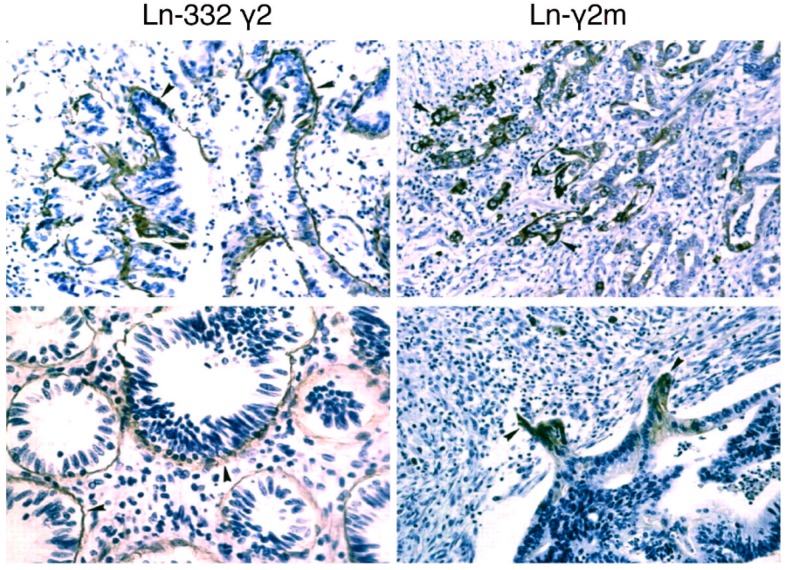
Detection of the γ2 chain of Ln-332 trimer and monomeric Ln-γ2 (Ln-γ2m) chain in gastric carcinoma in vivo. The γ2 chain of Ln-332 trimer was detected in the basement membranes of carcinoma tissues, whereas monomeric γ2 chain was selectively detected in the leading edge of carcinomas. Arrowheads, positive signal for the monomeric Ln-γ2 chain. Magnification: 200×. Figure 2 is modified from Koshikawa et al. [14].

Heterotrimeric Ln-332 expression is observed as a single layer on BMs in both normal epithelium and carcinoma tissues in vivo; however, Ln-332 expression is generally reduced in advanced tumors [11,12] (Figure 1 and Figure 2). Although the mechanism of Ln-332 down-regulation has not been fully elucidated, each laminin chain is observed either in monomeric or dimeric form in tumor-stage dependent manner. Thus, monomeric and/or dimeric laminin chains are believed to be specific biomarkers for malignant tumors [13]. In this review, we focus on the monomeric Ln-γ2 chain, which has been suggested to be involved in tumor cell invasion. The monomeric Ln-γ2 chain was originally identified in conditioned medium obtained from gastric carcinoma cells, and its expression was detected by immunohistochemistry in budding or disseminating tumor cells in gastric carcinoma tissues (Figure 2) [14]. Many lines of evidence have reported similar results in pancreatic, gastric, tongue, colorectal, lung, cervical, and esophageal carcinomas (Table 1) [14,15,16,17,18,19].

Similar to the γ2 chain of Ln-332 trimer, the monomeric Ln-γ2 chain is also cleaved by proteases. Membrane type-1 matrix metalloproteinase (MT1-MMP/MMP-14) cleaves domain III of Ln-γ2 chain, which contains a laminin-EGF-like motif and promotes malignant cancer progression via EGFR and its downstream signaling (Figure 1) [26].

## 3. Development of a Specific Antibody against Monomeric Ln-γ2 Chain

Since monomeric Ln-γ2 chain is believed to be a promising target for invasive cancers, previous studies have extensively reported the expression and localization of monomeric Ln-γ2 chain in cancer tissues [2,14,21,25,27,28,29]. For detecting monomeric Ln-γ2 chain by an immunoassay, two different laminin chain antibodies (Abs) (e.g., anti-Ln-α3 and Ln-γ2 mAbs) are required. For example, existence of the monomeric Ln-γ2 chain can be determined from Ln-α3 (negative) and Ln-γ2 (positive) immunostaining. The present immune method for monomeric Ln-γ2 detection only predicts its expression, since no antibody that specifically reacts with monomeric γ2, but not with the γ2 chain of Ln-332 trimer, is commercially available. Moreover, immunoassay with multiple antibodies is complicated and exhibits low detection sensitivity. Due to these major reasons, monomeric Ln-γ2 has not been applied in clinical cancer diagnosis yet.

**Figure 3:**
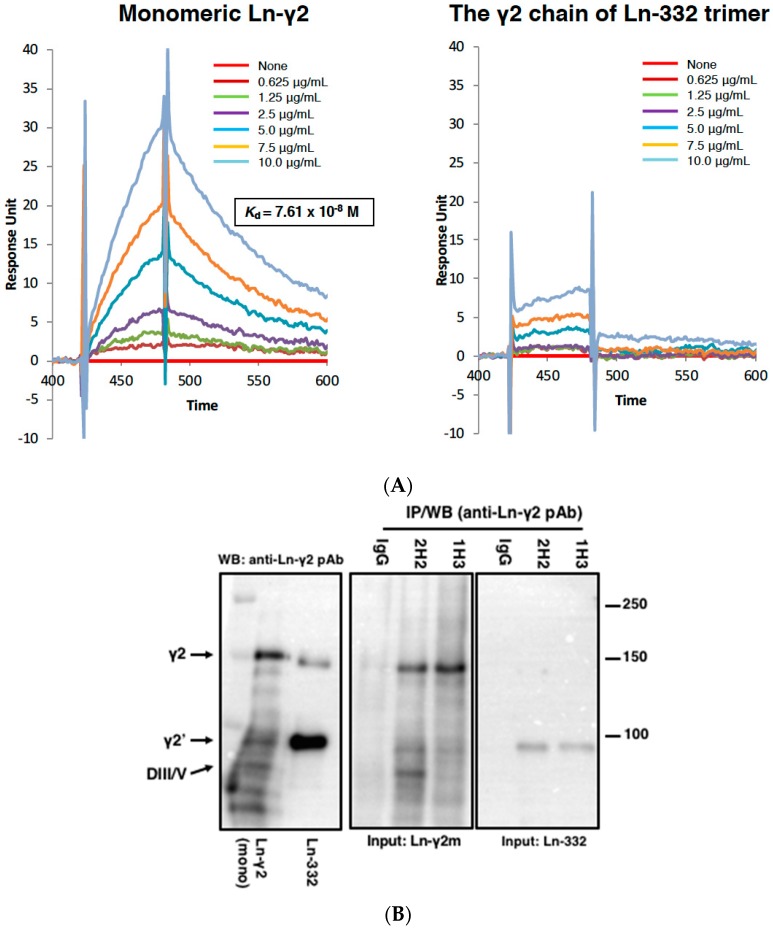

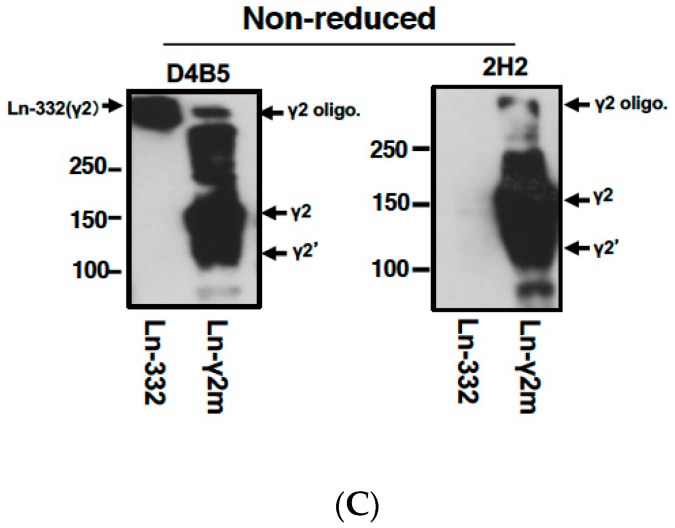
(**A**) The sensorgrams of SPR analysis for interactions between 2H2 mAb and monomeric Ln-γ2 (left), and 2H2 mAb and Ln-332 (right); 2H2 reacts with monomeric Ln-γ2 chain but not with the γ2 chain of Ln-332 trimer. (**B**) Immunoprecipitation (IP) of Ln-γ2 chain by 1H3 and 2H2 mAbs. Purified monomeric Ln-γ2 or Ln-332 (0.5 μg) protein was incubated with mouse immunoglobulin (IgG), 1H3 mAb, or 2H2 mAb (2 μg/mL each). Antibody-antigen complexes were then precipitated and subjected to western blotting (WB) using anti-Ln-γ2 polyclonal antibody (pAb). In addition to the intact form of Ln-γ2 (140 kDa), NH_2_-terminal (70 kDa) and COOH-terminal (100 kDa) fragments, processed by MMP-14 or mTLDs (Figure 1, right ②), are detected and indicated as γ2, DIII/IV, and γ2’, respectively. Left, sample proteins (monomeric Ln-γ2 and Ln-332) used for the assay were directly analyzed by WB using anti-Ln-γ2 pAb. Figure 3B is from Koshikawa et al. [30]. (**C**) Use of 2H2 and D4B5 mAbs in WB analysis. D4B5 is a commercially available anti-Ln-γ2 mAb. Purified monomeric Ln-γ2 (1 μg) and Ln-332 trimer (3 μg) proteins were separated by 7.5% SDS-PAGE and blotted on PVDF membranes under non-reducing conditions, and then detected either by D4B5 or 2H2 mAb. *Arrows,* Ln-γ2 homo-oligomer *(*γ2 oligo), monomer (γ2), its processed fragment (γ2’), and Ln-332.

To overcome this limitation, we generated specific antibodies against monomeric Ln-γ2, and subsequently isolated two different hybridoma clones (1H3 and 2H2 mAbs) using screening assays such as enzyme-linked immunosorbent assay (ELISA) and western blotting [30]. The equilibrium dissociation constant (*K*_d_) of 2H2 mAb for monomeric Ln-γ2 was determined to be 7.61 × 10^−8^ M using surface plasmon resonance (SPR), though the same for the γ2 chain of Ln-332 trimer was not detected (Figure 3A). Therefore, 2H2 mAb was established, for the first time, as a powerful tool for the specific detection of monomeric Ln-γ2, and could be used for immunoassays, including immunoprecipitation, ELISA, immunohistostaining, and western blotting (Figure 3B,C).

## 4. Detection of Ln-γ2 or Its N-Terminal Domain Fragment in Serum Specimens of Patients with Cancer

Katayama et al. established a sandwich ELISA for Ln-γ2 Domain IV–V fragment using a mAb against the DIV-V fragment [31,32], and evaluated it in serum specimens of patients with cancer. The sandwich ELISA revealed that patients with head and neck squamous cell carcinoma, hepatocellular carcinoma, bile duct carcinoma, gallbladder carcinoma, and pancreatic carcinoma with liver metastasis exhibited higher serum Ln-γ2 DIV-V fragment levels than healthy volunteers. Moreover, the study revealed that patients with benign digestive diseases also showed higher DIV-V levels than healthy volunteers. Furthermore, it was possible to monitor the lung epithelial repair process by measuring the serum Ln-γ2 DIV-V level in patients with acute lung injury [33]. Presumably, in sandwich ELISA, the mAb reacts with DIV-V fragments derived from both heteromeric and monomeric Ln-γ2 chains and cannot distinguish between patients with cancer and those with inflammatory diseases that release Ln-332 from BMs through ECM degradation.

A recent study by Kosanam et al. evaluated Ln-γ2 in the serum of patients with pancreatic carcinoma using a commercially available ELISA kit [24]; the serum Ln-γ2 level in patients with pancreatic carcinoma was found to be significantly elevated compared to that in patients with chronic pancreatitis and healthy volunteers in three cohort studies. This result strongly suggests that monomeric Ln-γ2 might be a potential candidate biomarker for cancer.

## 5. Establishment of an Automated Chemiluminescent Immunoassay (CLIA)

**Figure 4:**
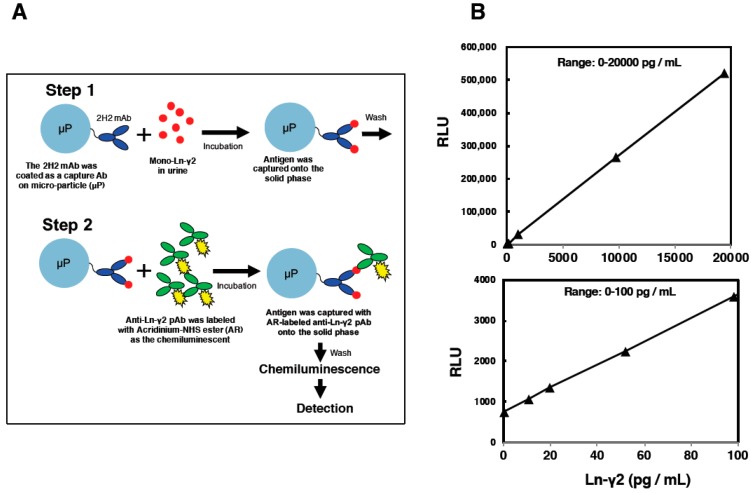
(**A**). Detection method for Chemiluminescent immunoassay (CLIA) (**B**), and its standard curve at different ranges using recombinant monomeric Ln-γ2 protein. RLU; Relative Light Unit.

Based on previous findings, we established a detection system using a chemiluminescent immunoassay (CLIA) for estimating serum monomeric Ln-γ2 levels using 2H2 mAb, and applied it for clinical cancer diagnosis. The CLIA is a highly sensitive protein detection system with low background, compared to sandwich ELISA, and is a common biomarker assay in clinical diagnosis (Figure 4A). The 2H2 mAb was introduced in CLIA assay to eliminate cross-reaction with the γ2 chain of Ln-332 trimer directly [22,34]. The CLIA assay with 2H2 mAb can reduce false-positive reactions and enhance the diagnostic accuracy. Indeed, a standard curve using recombinant monomeric Ln-γ2 protein shows that the CLIA can measure from 0 to 20,000 pg/mL of the protein (Figure 4B). The lower detection limit is 10 pg/mL, and serum monomeric Ln-γ2 levels in healthy volunteer’s average to 44.3 ± 17.6 pg/mL (mean ± SD) [22]. These data collectively indicate that measurement efficiency of the CLIA exceeds that of sandwich ELISA (Table 2). Therefore, we evaluated the serum monomeric Ln-γ2 level in serum specimens of patients with digestive cancers, using CLIA, and found it to be a promising biomarker for early detection of hepatocellular carcinoma (HCC), as described below.

## 6. Hepatocellular Carcinoma Surveillance and Biomarkers

There has been remarkable development in therapeutic options for HCC recently. Nevertheless, curative options are only feasible in case of early diagnosis. Screening programs in an increased-risk population could lead to more frequent detection of HCC at early stages and reduce HCC mortality [35]. The subjects of regular HCC surveillance include patients with chronic hepatitis B virus (HBV), chronic hepatitis C virus, and non-viral liver cirrhosis. Tests that can be used in HCC surveillance include serological and imaging examinations. Ultrasonography is the most widely used method for HCC surveillance. In Taiwan, residents aged between 45 and 69 years, with a high prevalence of hepatitis B surface antigen (HBsAg), were invited to community-based abdominal ultrasonography screening for HCC, followed by subsequent reduction in HCC-related mortality compared to that in those who were not invited [36]. The American Association for the Study of Liver Diseases has recommended six monthly ultrasonography screening for HCC [37]. Ultrasonography is not invasive, but is relatively expensive and operator- and patient-dependent. In contrast, serological biomarkers can be used at relatively low costs, without any burden to the patient. α-fetoprotein (AFP) is the most frequently used biomarker for HCC worldwide. Screening with six monthly AFP assays in HBV-positive individuals resulted in earlier diagnosis of HCC, but did not affect five-year survival [38]. Randomized controlled trial indicated that biannual screening with combination of AFP and ultrasound reduced HCC-mortality in individuals with HBV infection or history of chronic hepatitis. Patients with early and surgically respectable stages of HCC were found significantly more often in the screened group than in the control [35]. Surveillance program of patients with liver cirrhosis combined AFP and ultrasound to prolong survival rate of patients with HCC-mortality [39]. Thus, the Japan Society of Hepatology has recommended surveillance with six monthly ultrasonography and biomarker assays every three to four months for high-risk populations [40].

In surveillance, biomarkers are used to complement imaging tests, alone or in combination. AFP and prothrombin induced by Vitamin K Absence II (PIVKA-II), also known as des-gamma-carboxy prothrombin, are the most commonly used biomarkers for HCC surveillance. Currently, the recommended clinical cut-off values are 20 ng/mL for AFP and 40 mAU/mL for PIVKA-II. A case-control study in patients with chronic HBV infection with or without HCC, showed sensitivity 57.5% and specificity 88% for AFP, and sensitivity 51.9% and specificity 97% for PIVKA-II [41]. A recent systematic review has indicated sensitivity 59% and specificity 86% for AFP, and sensitivity 63% and specificity 91% for PIVKA-II [42]. AFP is the most commonly used biomarker; however, small HCCs are not always associated with elevated AFP in serum, and even among large HCCs, only about 80% show elevated AFP. Therefore, an appropriate combination of these markers might increase sensitivity. Combination of AFP and PIVKA-II increased the sensitivity to 78.3% [41]. Among early stage HCC, the receiver operating characteristic area under curve (ROC curve AUC) of PIVKA-II, AFP, and combination of both markers were 0.84, 0.68, and 0.83, respectively; the combination of PIVKA-II and AFP did not seem to be better than PIVKA-II in detecting early stages of HCC [42]. Development of more effective biomarker combination would be required for early HCC diagnosis.

Biomarker monitoring is also useful in detecting HCC recurrence after therapeutic intervention. Positive AFP and PIVKA-II status became negative at 6 months post-hepatectomy in 80.3% and 99.6% patients, respectively. AFP and PIVKA-II levels in patients showing recurrence in ≤6 months correlated with the levels measured before hepatectomy [43]. Postoperative AFP level is, therefore, a useful tool for predicting recurrence after curative hepatectomy. A positive level of post-operative AFP might suggest a site of residual viable cancer [44].

## 7. Serum Monomeric Ln-γ2 as a Novel Biomarker for Hepatocellular Carcinoma

Although heterotrimeric Ln-332 is not detected in normal hepatic tissues [29], increased expression of monomeric Ln-γ2 in HCC tissue has been shown to be associated with a more proliferative and metastatic phenotype [23]. In HCC, invasive tumor cells secrete TGF-β1, which triggers invasiveness and motility in Ln-332 by inducing the expression of the transmembrane integrin receptor α3β1. Ln-332 upregulates the expression of the transcriptional repressors Snail and Slug, which induce the EMT together with TGF-β1, and downregulating E-cadherin, followed by translocation of β-catenin to the nucleus [45]. According to a previous report, indeed all HCC cells tested expresses Ln-γ2, MT1-MMP and MMP2. It is believed that the Ln-γ2 processing presumably occurs and plays roles in their growth, motility and invasiveness through the EGFR activation [46].

**Figure 5:**
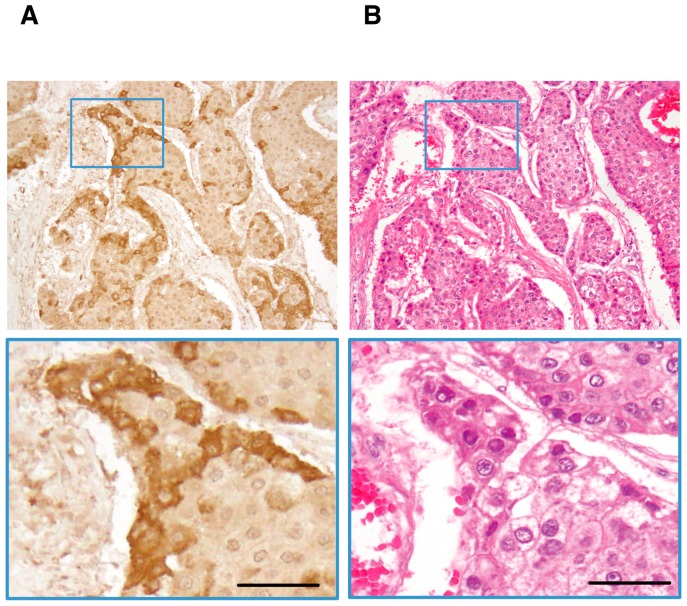
(**A**). Immunohistochemical staining of Ln-γ2 in HCC. A representative example of positive staining for Ln-γ2. (**B**). HE staining. Scale bars, 50 μm. Figure 5 is modified from Kiyokawa et al. [22].

As shown in Figure 5, cytoplasmic staining of Ln-γ2 was observed in surgically removed HCC nodules. Ln-γ2 immunoreactivites are preferentially observed in marginal-moderately to poorly differentiated parts of HCC nodules [22], as is observed in gastric carcinoma tissue [14]. Monomeric Ln-γ2 is also expressed in human HCC-derived cell lines. Recent report indicated that Ln-γ2 is highly found in HCCs expressing the biliary marker keratin 19 [47]. Aberrant activation of Wnt/β-catenin signaling is a common genetic abnormality in human HCC [48]. Previous studies on the mechanism of Ln-γ2 expression had demonstrated that its gene and protein expression are up-regulated in gastric and colon cancer cells by transcriptional factor 4 (TCF4)/β-catenin and/or Wnt-5a [20,49]. Moreover, a study on comparative genomic hybridization (CGH), using cancer specimens, demonstrated that copy number of the gene encoding Ln-γ2 is frequently increased in both early and advanced stages of hepatocellular carcinoma (HCC) and lung squamous cell carcinoma [27]. Furthermore, expression of Ln-332 was seen to be lost in many types of cancers due to gene methylation [50,51,52,53,54]. These reports together support the increased expression of monomeric Ln-γ2 in HCC tissue.

**Figure 6:**
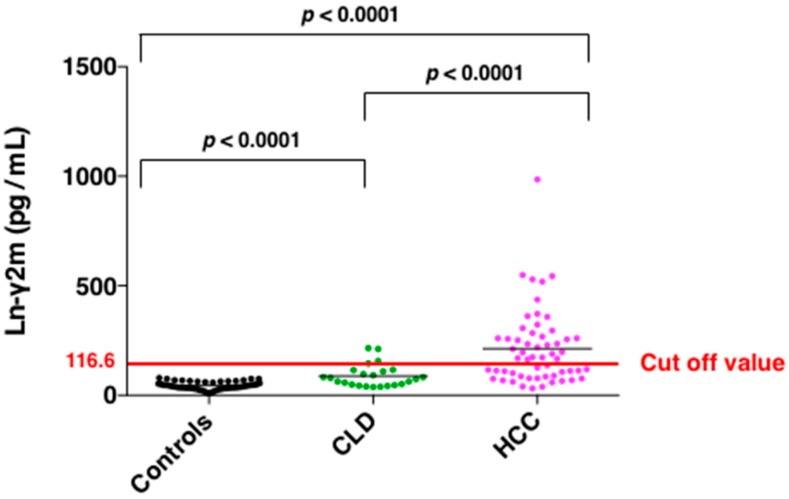
Scatter plot of Ln-γ2 concentrations determined in serum samples from healthy subjects (*n* = 52), patients with CLD (*n* = 24), and patients with HCC (*n* = 57). The optimal cutoff value for Ln-γ2 to distinguish between HCC and non-malignant CLD is 116.6 pg/mL. Figure 6 is modified from Kiyokawa et al. [22].

**Figure 7:**
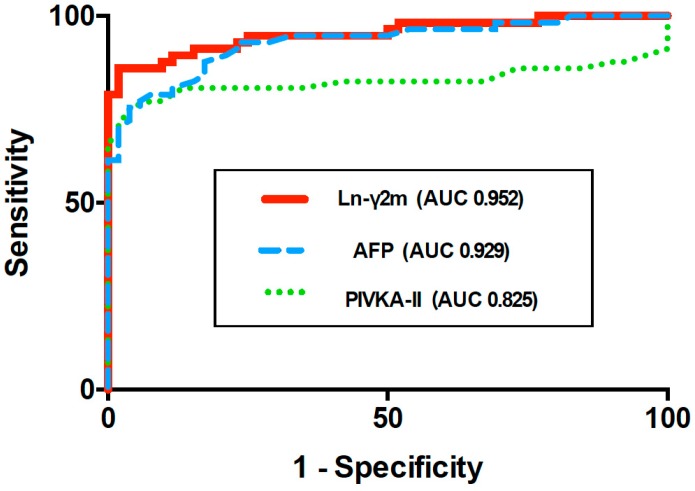
ROC curve AUC of monomeric Ln-γ2, PIVKA-II, and AFP in patients with HCC versus healthy volunteers. Figure 7 is modified from Kiyokawa et al. [22].

Since monomeric Ln-γ2, rather than heterotrimeric Ln-γ2, is expressed preferentially in HCC nodules, we have evaluated the diagnostic value of monomeric Ln-γ2, AFP, and PIVKA-II in sera from patients with HCC and chronic liver diseases (CLD) using the above-mentioned automated CLIA along with ARCHITECT [22]. A significant increase in monomeric Ln-γ2 levels was observed in patients with HCC compared to patients with CLD and healthy volunteers (Figure 6). ROC curve AUC of monomeric Ln-γ2, PIVKA-II, and AFP were 0.952, 0.825, and 0.929, respectively, when comparing healthy volunteers and patients with HCC. The discriminative ability of monomeric Ln-γ2 significantly surpassed that of PIVKA-II, and was as effective as AFP (Figure 7). When discriminating patients with non-malignant CLD from those with HCC, ROC curve AUC of monomeric Ln-γ2, PIVKA-II, and AFP were 0.793, 0.845, and 0.788 respectively [22]. Therefore, serum monomeric Ln-γ2 seems to be more effective than AFP in differentiating patients with HCC from those with CLD.

**Figure 8:**
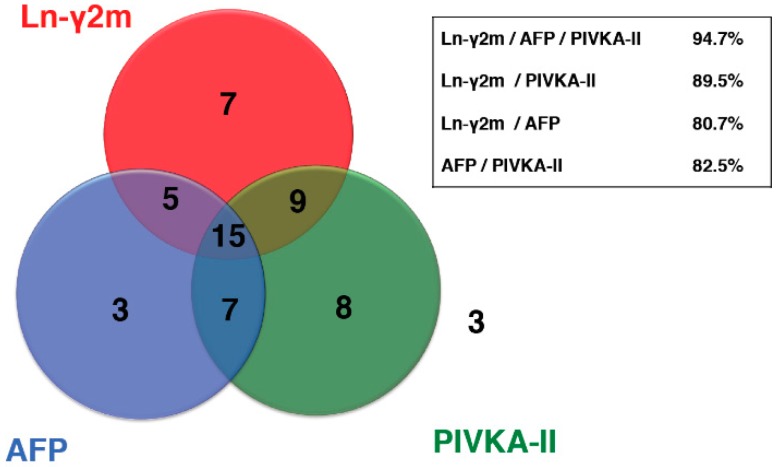
Serum monomeric Ln-γ2 levels were measured in 57 patients with HCC. HCC positive rates, obtained when combining two biomarkers, were compared. Three patients were negative for all three biomarkers. HCC detection rates for the combination of Ln-γ2 and PIVKA-II, Ln-γ2 and AFP, and PIVKA-II and AFP, were 89.5% (51/57), 82.5% (47/57), and 80.7% (46/57), respectively. A combination of all three markers was detected in 54/57 patients (94.7%). Figure 8 is modified from Kiyokawa et al. [22].

In addition, the positivity rate in patients with HCC for the combination of Ln-γ2 and PIVKA-II was 89.5%, whereas that for monomeric Ln-γ2 and AFP was 80.7%, and for PIVKA-II and AFP was 82.5%. The combination of Ln-γ2 and PIVKA-II seemed to make a more sensitive pair of biomarkers compared to a conventional marker (Figure 8).

**Figure 9:**
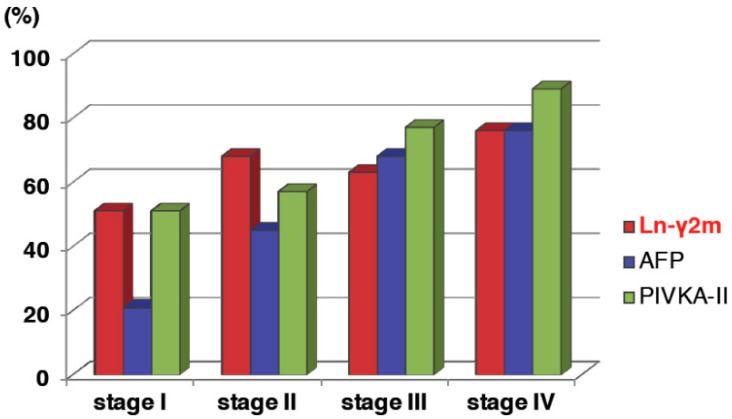
Comparison of the biomarker-positive rate in HCC by tumor stages. Figure 9 is modified from Kiyokawa et al. [22].

Increase of monomeric Ln-γ2 levels is observed with the stepwise progression of CLD, and according to tumor stages. The optimal cutoff value for Ln-γ2 to distinguish between HCC and nonmalignant CLD was 116.6 pg/mL. Positivity rate of monomeric Ln-γ2 in patients with HCC for each TMN stage was 50% in stage I, 67% in stage II, 62% in stage III, and 75% in stage IV, respectively, whereas that of AFP was 20% in stage I, 44% in stage II, 67% in stage III, and 75% in stage IV, respectively, and of PIVKA-II was 50% in stage I, 56% in stage II, 76% in stage III, and 88% in stage IV, respectively (Figure 9) [32]. Positivity rate of monomeric Ln-γ2 is clearly higher than AFP and comparable to PIVKA-II. Among patients with early-stage HCC (T1 or T2; the T factor includes three criteria: solitary tumor, maximum tumor diameter < 2 cm and no vascular invasion. T1 meets all three criteria, T2 meets two of the three criteria), the positivity rates of monomeric Ln-γ2 may be higher than AFP or PIVKA-II. Taken together, these results indicate the potential clinical applicability of monomeric Ln-γ2 for the detection of early-stage HCC. Besides being a diagnostic marker, it would be of particular interest, in the future, to examine the potential of serum monomeric Ln-γ2 as a biomarker to monitor therapeutic effects.

## 8. Conclusions

Monomeric Ln-γ2 was identified as a biomarker, which is specifically expressed on the cancer invasion front. Although monomeric Ln-γ2 has long been of interest as a potential biomarker for cancer diagnosis owing to its unique biological features, development of an assay system for Ln-γ2 single chain faced many obstacles, considering that Ln-γ2 is a part of Ln-332 trimer and most antibodies that react with Ln-γ2 chain also recognize the Ln-332 trimer. We have therefore developed mAbs that specifically detect monomeric Ln-γ2. Previous research has indicated important roles of Ln-γ2/Ln-322 in pathophysiology of HCC. Using this tool, we have thus further developed highly sensitive CLIA for serum monomeric Ln-γ2. Serum monomeric Ln-γ2 may be considered a clinically available biomarker for HCC surveillance. Moreover, the combination of monomeric Ln-γ2 and PIVKA-II may become a sensitive tool for clinical diagnosis of HCC at early stages, hence preventing HCC-related deaths.

## Figures and Tables

**Table 1:** Reported studies of expression of Ln-γ2 in cancer of gastrointestinal tract.

Organ	Detection Rate by IHC	Detection Rate by Serological Assay	References
esophagus	44% (44/100)	N/A	[18]
stomach	23% (35/153)	N/A	[20]
colon	65% (29/39)	N/A	[21]
liver	63% (25/40)	CLIA, 63% (36/57)	[22,23]
pancreas	53% (8/15)	ELISA, 72% (36/50)	[16,24]
biliary tract	57% (35/61)	N/A	[25]

N/A; not available.

**Table 2:** Comparison of measurement efficiency between LISA and CLIA.

	ELISA	Automated CLIA
Dynamic range	Narrow *1	Wide *2
Detection sensitivity	Low	High
Diagnostic accuracy	Low	High
Background	High	Low
Throughput	Low	High
Reaction time	> 2.5 h	30 min

*1: Note that dynamic range of ELISA is 15–4000 pg/mL *2, and CLIA is 10–20,000 pg/mL.

## Abbreviations

AFP	α-fetoprotein
BM	basement membrane
PIVKA-II	prothrombin induced by Vitamin K Absence II
CLIA	chemiluminescent immunoassay
CLD	chronic liver disease
ECM	extracellular matrix
HBV	hepatitis B virus
HCC	hepatocellular carcinoma
HCV	hepatitis C virus
IP	Immunoprecipitation
Ln-332	laminin 332
Ln-γ2	laminin-γ2
mAb	monoclonal antibody
pAb	polyclonal antibody
ROC curve AUC	receiver operating characteristic area under curve
